# Proteomic analysis to identify markers for response to neoadjuvant treatment in esophageal and gastroesophageal cancer

**DOI:** 10.1002/cnr2.1489

**Published:** 2021-08-05

**Authors:** Oran Zlotnik, Tal Goshen‐Lago, Riad Haddad, Baruch Brenner, Yulia Kundel, Irit Ben‐Aharon, Hanoch Kashtan

**Affiliations:** ^1^ Department of Surgery Rabin Medical Center Petach Tikva Israel; ^2^ Sackler Faculty of Medicine Tel‐Aviv University Tel‐Aviv Israel; ^3^ Oncology Division Rambam Health Care Campus Haifa Israel; ^4^ Rappaport Faculty of Medicine Technion Haifa Israel; ^5^ Department of Surgery Carmel Medical Center Haifa Israel; ^6^ Institute of Oncology, Davidoff Center, Rabin Medical Center Petach Tikva Israel

**Keywords:** biomarkers, esophageal cancer, gastroesophageal cancer, proteomics

## Abstract

**Background:**

Esophageal cancer represents a global challenge. Despite significant evolution of treatment protocols in the past decade, recurrence rates are still high and survival rates are poor. Current treatment paradigm for localized gastroesophageal junction (GEJ) carcinoma remains to be further elucidated as for the role of neoadjuvant chemoradiation versus perioperative chemotherapy.

**Aim:**

To identify biomarkers for response to chemoradiation in esophageal and gastroesophageal cancer, we performed an in‐depth proteomic analysis of esophageal and gastroesophageal tumors, to describe differences in pathway activation between patients with favorable and poor prognosis following neoadjuvant chemoradiation.

**Methods:**

Patients with locally advanced esophageal and gastroesophageal cancer following neoadjuvant chemoradiation were included in the cohort. The study cohort was dichotomized into two groups of patients, named “favorable prognosis” and “poor prognosis” according to the postoperative disease‐free interval. We performed a mass spectrometry analysis of proteins extracted from the malignant regions of surgical specimens and analyzed data from electronic medical records. Clinical data was correlated with differences in protein expression between patient with a favorable and poor prognosis using validated gene expression pathways.

**Results:**

The study included 35 patients with adenocarcinoma. All patients in this cohort had esophageal adenocarcinoma. Patients median age was 62 years. Twenty‐five (71.3%) patients underwent neoadjuvant chemoradiation, and 28.7% underwent neoadjuvant chemotherapy only. A proteomic analysis of our cohort identified 2885 proteins. Enrichment levels of 98 of these proteins differed significantly between favorable and poor prognosis cohorts in patients who underwent neoadjuvant chemoradiation (*p* < .05) but not in patients who underwent neoadjuvant chemotherapy. The favorable prognosis patients group analysis exhibited differential enrichment of 87 proteins related to cellular respiration and oxidative phosphorylation pathways as well as proteins of the RAS oncogene family.

**Conclusion:**

In this study we identified differential enrichment of pathways related to oxidative phosphorylation and RAS oncogene pathway in esophageal cancer patients with a favorable response to chemoradiation. Following further validation, our findings may portray potential surrogate signature of biomarkers based upon these pathways.

## 1 INTRODUCTION

Esophageal cancer represents a global challenge due to its rising incidence^1^ and dismal prognosis.^2^ Standard treatments of locally advanced esophageal cancer consist of neoadjuvant chemoradiotherapy or chemotherapy followed by surgery. Despite significant evolution of treatment protocols in the past decades, recurrence rates are still high and survival rates are poor.^3^

Gastroesophageal junction tumors were previously shown to be effectively treated with neoadjuvant chemoradiation in the Landmark RTOG 8501 trial.^4^ In this trial a regimen of cisplatin and 5‐fluorouracil (5‐FU) combined with radiotherapy improved patient outcome significantly. The more recent Cross trial^5^ which utilized neoadjuvant carboplatin and paclitaxel combined with radiation therapy also demonstrated a significant increase in 5‐year overall survival in patients with esophageal or gastroesophageal junctional (GEJ) cancer. Long‐term follow‐up of the Cross‐trial participants confirmed the survival benefit.^6^

Other milestone trials have demonstrated that perioperative chemotherapy alone may also provide a significant survival advantage for gastroesophageal junction cancer patients. The most significant were the MAGIC trial^7^ (with 12% GEJ tumors and 15% lower esophageal tumors) and the French FFCD trial^8^ (64% with GEJ tumors and 11% lower esophageal tumors). The recent FLOT4 study has demonstrated even further improved pathological response rates and improved median overall survival^9^ and thus replaced former regimens as the current standard of care. Current treatment paradigm for localized gastroesophageal junction (GEJ) carcinoma remains to be further elucidated as for the role of chemoradiation versus perioperative chemotherapy. Stratification of gastroesophageal patients into subgroups according to biomarkers may allow more informed and personalized choices of neoadjuvant chemoradiation or chemotherapy protocols.

As part of a global effort to search for biomarkers and improve the biological understanding of esophageal and gastroesophageal cancers, Kim et al^10^ has characterized the genomic landscape of 164 esophageal carcinomas from western and eastern populations and categorized them into molecular subtypes. This study demonstrated how esophageal squamous cell carcinoma has greater similarity to head and neck cancers than to esophageal adenocarcinoma, while esophageal adenocarcinoma had greater resemblance to gastric adenocarcinoma. These findings argue against formerly adopted approaches to include both squamous and esophageal carcinoma into combined clinical trials and treatment protocols and emphasize the need to stratify patients into histopathological and molecular subgroups.

Proteomics analysis is a powerful tool to identify biomarkers for response to treatment. As part of the effort to subcategorize gastroesophageal and identify biomarkers for response to treatment, previous esopahgeal cancer proteomics studies have identified markers for adverse outcome, such as GLUT1‐sialyl‐Tn antigen which is also expressed in other gastrointestinal cancers,^11^ as well as differentially enriched proteins related to mitochondrial apoptosis, and regulation of angiogenesis.^12^ However, most of these studies have compared esophageal cancer cells with healthy esophageal tissue cells. In the current study, we aimed to find proteomic biomarkers to predict a favorable response to neoadjuvant chemoradiation. We compared the proteomic profile of patients who underwent neoadjuvant chemoradiation to that of patients who underwent neoadjuvant chemotherapy and no radiation. Our aim was to identify a proteomic signature that will predict a favorable response to neoadjuvant chemoradiation in gastroesophageal cancer patients.

## 2 METHODS

### 2.1 Patients

Consecutive patients with esophageal and gastroesophageal junction cancer who underwent neoadjuvant therapy followed by surgery were identified from a prospectively maintained surgical databases at our institution from 2008 to 2015, and for whom formalin fixed frozen paraffin embedded (FFPE) surgical samples were available were included in the study. The treatment plan for each patient was determined during a weekly multidisciplinary meeting.

Clinical data included demographics, preoperative tumor staging, lab results, tumor markers, type of neoadjuvant treatment, surgical techniques, complications and outcome, postoperative adjuvant treatment, pathologic tumor staging, and long‐term follow‐up. These data were retrieved from electronic medical records.

Patients were dichotomized into two groups, named “favorable prognosis” (FP) and “poor prognosis” (PP) based upon the time interval from surgery to disease recurrence. Patients who had no recurrence of disease at least 1 year following surgery were defined as “favorable prognosis” and patients that demonstrated tumor recurrence within 1 year of surgery or less were defined as “poor prognosis.”

### 2.2 Proteomics

Pathological slides from all patients in the cohort were reviewed by a pathologist from our institution, that also chose the relevant tumor areas for analysis. These areas were dissected and used for proteomics analysis.

Key components of the quantitative proteomics approach were employed, allowing the quantitation of targeted proteins from FFPE tumor biopsy.

FFPE samples from different patients under different treatments, were micro‐dissected to isolate and collect only the tumor cells of interest for analysis. Paraffin embedded tumor tissue sections (10 μm), were deparafinized using *n*‐Hexan for 30′ (shaking, RT) followed by methanol addition and phase separation. Following centrifugation, the upper phase was discarded, and the lower phase was dried, washed once with 50% ethanol and dried again. Each pellet was then suspended with 60ul of Urea buffer containing: 8 M Urea, 400 mM Ammonium bicarbonate, and 10 mM DTT and incubated for 2 h at 600 C (shaking). Samples were then sonicated (5′, [10/10 on/off pulses, 90% energy, Sonics Vibra‐Cell] and incubated again for 30′ at 600°C (shaking) for full protein reduction. Protein samples were then modified with 40 mM iodoacetamide in 100 mM ammonium bicarbonate (in the dark, RT, 30′) and digested in 2 M Urea, 100 mM ammonium bicarbonate with modified trypsin (Promega) overnight at 370°C at a 1:50 enzyme‐to‐substrate ratio. A Four‐hour trypsin digestion was performed at 370 C with a 1:100 enzyme‐to‐substrate ratio. The tryptic peptides were desalted using C18 tips (Top tip, Glygen) dried and re‐suspended in 0.1% Formic acid.

The peptides were resolved by reverse‐phase chromatography on 0.075 X 180‐mm fused silica capillaries (J&W) packed with Reprosil reversed phase material (Dr Maisch GmbH, Germany). The peptides were eluted with linear 180 min gradient of 5%–28% 15 min gradient of 28%–95% and 25 min at 95% acetonitrile with 0.1% formic acid in water at flow rates of 0.15 μl/min. Mass spectrometry was performed by Q Exative HFX mass spectrometer (Thermo) in a positive mode (m/z 300–1800, resolution 120 000 for MS1 and 15 000 for MS2) using repetitively full MS scan followed by collision induces dissociation (HCD, at 27 normalized collision energy) of the 30 most dominant ions (>1 charges) selected from the first MS scan. The AGC settings were 3 × 10^6^ for the full MS and 1 × 10^5^ for the MS/MS scans. The intensity threshold for triggering MS/MS analysis was 1 × 10^4^. A dynamic exclusion list was enabled with exclusion duration of 20 s.

The mass spectrometry data was analyzed using the MaxQuant software 1.5.2.8^13^ for peak picking and identification using the Andromeda search engine, searching against the human proteome from the Uniprot database with mass tolerance of 6 ppm for the precursor masses and 20 ppm for the fragment ions. Oxidation on methionine and protein N‐terminus acetylation were accepted as variable modifications and carbamidomethyl on cysteine was accepted as static modifications. Minimal peptide length was set to six amino acids and a maximum of two miscleavages was allowed. The data was quantified by label free analysis using the same software. Peptide‐ and protein‐level false discovery rates (FDRs) were filtered to 1% using the target‐decoy strategy. Protein table were filtered to eliminate the identifications from the reverse database, and common contaminants and single peptide identifications.

Statistical analysis of the identification and quantization results was done using Perseus 1.6.2.2.^14^ *T*‐test was done, and differential proteins were proteins with *p* value < .1 with at least twofold change (>1 or −1> at the difference).

## 3 RESULTS

Thirty‐five patients included in the study cohort underwent neoadjuvant therapy followed by surgery. Twenty‐five (71.3%) of these patients underwent neoadjuvant chemoradiation, and 10 patients (28.7%) underwent neoadjuvant chemotherapy only. All the patients in the study cohort had esophageal adenocarcinoma. There was a male predominance (85.7%), and the median age was 62 years. Seventeen (48.5%) patients were defined as favorable prognosis (no recurrence for 1 year after surgery) and 18 (51.5%) as poor prognosis (recurrence within less than 1 year after surgery). Tumors located in the gastroesophageal junction were predominant in both cohorts. The type of surgery performed in most patients (*N* = 29, 82.8%) was minimally invasive three field esophagectomy with cervical anastomosis. Clinical and preoperative date of the entire cohort are summarized in Table 1.

**TABLE 1:** Clinical characteristics of all patients

Characteristics	Patients
Age, median (range)^a^	62 (39–84)
Adenocarcinoma	35/35
Squamous cell carcinoma	0/35
Gender	
Male	*N* = 30/35 (85.7%)
Female	*N* = 5/35 (14.2%)
**Favorable prognosis** ^**b**^	*N* = 17/35 (48.5%)
Tumor location	
Esophageal tumors	*N* = 3/17 (17.6%)
EGJ tumors	*N* = 14/17 (82.3%)
Preoperative treatment	
Chemotherapy + Radiation	*N* = 15/17 (88.2%)
Chemotherapy	*N* = 2/17 (11)
**Poor prognosis** ^**c**^	*N* = 18/35 (51.5%)
Tumor location	
Esophageal tumors	*N* = 6/18 (33.3%
EGJ tumors	*N* = 12/18 (66.6%)
Preoperative treatment	
Chemotherapy + Radiation	*N* = 10/18 (55.5%)
Chemotherapy	*N* = 8/18 (44.4%)8

^a^ These data relate to all the patients in the study cohort.

^b^ These data relate to patients in the favorable prognosis group (no evidence of recurrence within 1‐year post surgery.

^c^ These data relate to patients in the poor prognosis group (recurrence within 1‐year after surgery).

Proteomic analysis identified 2885 proteins. Relative enrichment levels of proteins were compared between the favorable prognosis and the poor prognosis cohorts. This analysis was performed separately for patients who were treated with neoadjuvant chemoradiation and for patients treated with neoadjuvant chemotherapy. In patients who were treated with chemoradiation, enrichment levels of 98 proteins differed significantly (*p* < .05) between patients with a favorable and poor prognosis. In patients who underwent chemotherapy only, enrichment level of these 98 proteins did not differ significantly between favorable and poor prognosis.

The clinical characteristics and outcome of patients who underwent neoadjuvant chemoradiation (25 patients) are summarized in Table 2. The median overall survival for patients with a favorable prognosis following chemoradiation cohort was 3.8 years compared with 1.2 years for patients with a poor prognosis following chemoradiation. Patient who received neoadjuvant chemoradiation and belonged to the poor prognosis cohort had higher rates of lymph node metastasis, higher rates of signet ring features and lower rates of complete pathological.

**TABLE 2:** Patients treated with chemoradiation—Clinical characteristics and outcome

	Favorable prognosis^a^ (*N* = 15)	Poor prognosis^b^ (*N* = 10)
Tumor location		
Esophageal	*N* = 3 (20%)	*N* = 4 (40%)
Gastroesophageal junction	*N* = 12 (80%)	*N* = 6 (60)
Pathological lymph nodes on preoperative imaging	*N* = 8 (53.3%)	*N* = 6 (60%)
Chemotherapy protocol		
Cisplatin+5fu	*N* = 11 (73.3%)	*N* = 7 (70%)
Carboplatin+ paclitaxel	*N* = 4 (26.6%)	*N* = 3 (30%)
Patients with lymphovascular invasion	*N* = 2 (13.3%)	*N* = 2 (20%)
Patients with pathological lymph node involvement	*N* = 5 (33.3%)	*N* = 7 (70%)
Signet ring cell features	*N* = 2 (13.3%)	*N* = 4 (40%)
Complete pathological response	*N* = 2 (13.3%)	*N* = 1 (10%)
Overall survival, median (range)	3.8 years (1.9–5.8)	1.3 years (0.3–2.2)

^a^ These data relate to patients in the favorable prognosis group (no evidence of recurrence within 1‐year after surgery).

^b^ These data relate to patients in the favorable prognosis group (recurrence within 1‐year after surgery).

**FIGURE 1:**
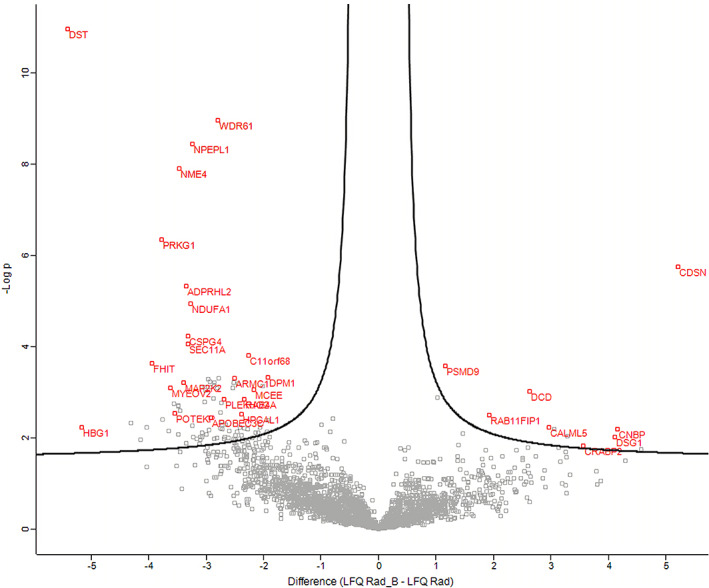
Protein scatter plot. This graph depicts a representative graph of label free quantification (LFQ) of proteins in favorable and poor prognosis cohorts on a logarithmic scale. The proteins with the most significant differential enrichment in the favorable prognosis group appear on the left side of the curve, and proteins with the most significant differential enrichment in the poor prognosis group appear on the right side of the curve

**FIGURE 2:**
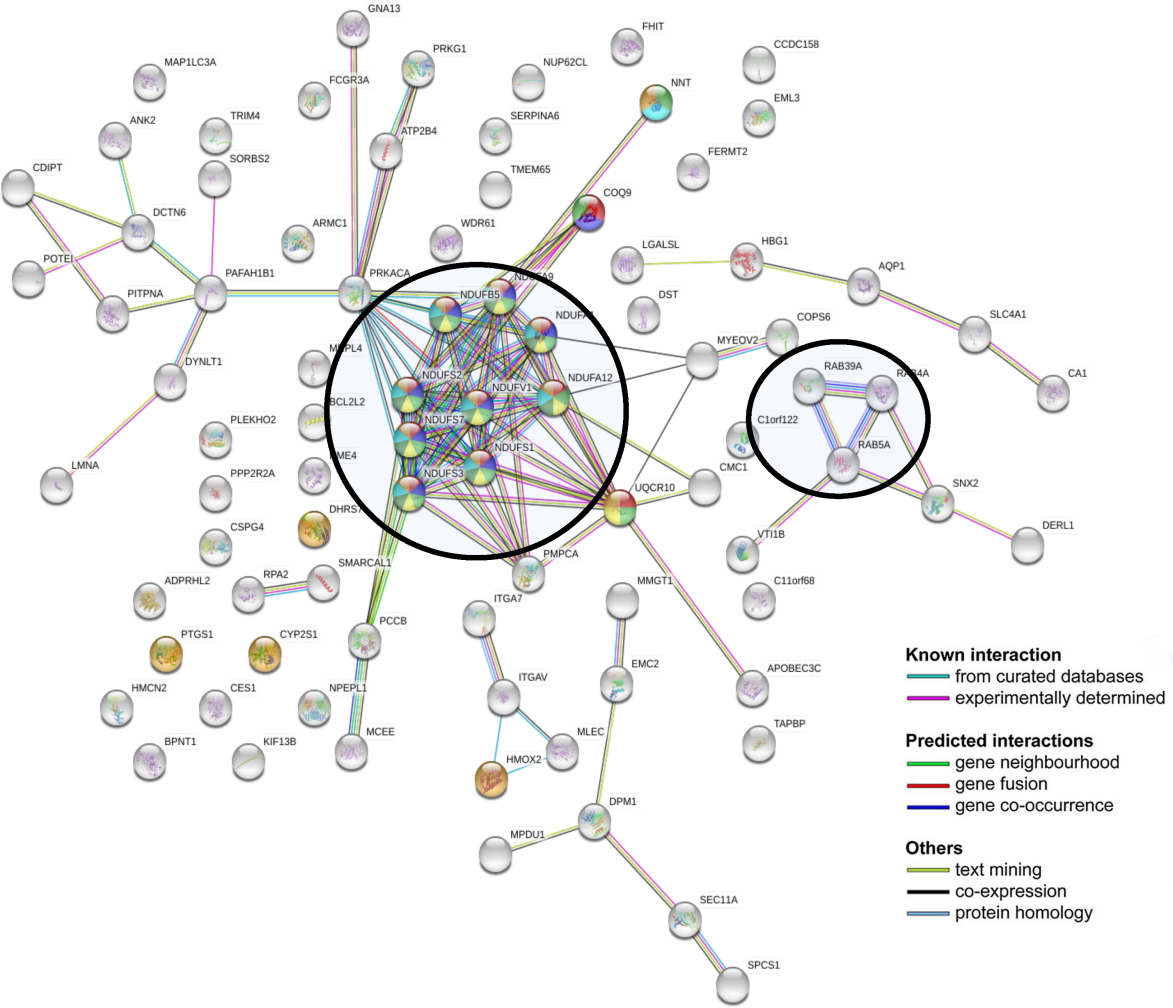
Representation by STRING software of differentially abundant proteins overexpressed in the favorable response group (https://string‐db.org). The nature of the interactions is shown at the bottom of the figure and lists the known interactions (from curated database, experimentally determined), the predicted interactions (gene neighberhood, gene fusion, gene co‐occurrence) and others (text mining, co‐expression, protein homology). Significantly overexpressed protein interactions were found in favorable prognosis versus poor prognosis with (A) NDUF proteins mitochondrial respiratory chain Complex 1 proteins. (B) RAB39A, RAB4A and RAB5A

Of the 98 proteins previously mentioned, 87 proteins had a significantly differential enrichment levels in patients with a favorable prognosis following chemoradiation. Analysis of the interactions between these 87 differentially enriched proteins was performed utilizing the STRING database.^15^ This analysis revealed statistically significant enrichment of biological processes related to cellular respiration, mitochondrial ATP synthesis coupled electron transport, organophosphate metabolic processes, and mitochondrial respiratory chain complexes assembly. Figure 1 illustrates protein expression following chemoradiation in the favorable prognosis and poor prognosis cohorts in a volcano plot. The protein interactions and pathway analysis enriched in the favorable prognosis group are illustrated in Figure 2. One group of interacting proteins identified in this analysis were the NDUF proteins which belong to mitochondrial respiratory Complex I, the largest component of the mitochondrial oxidative phosphorylation system. Another group of interacting proteins were RAB39A, RAB4A and RAB5 proteins which are members of the RAS oncogene family. RAB4A promotes local invasion and distant metastasis of tumor cell lines, and RAB5A is associated with lymphatic and vascular invasion.

In the poor prognosis cohort, 11 proteins had significantly differential enrichment levels. These proteins were—CDSN, MYH2, DSG1, CNBP, ADSSL1, CRABP2, LEMD2, DCD, CALML5, RAB11FIP, RAB11FIP2 and PSMD9. Although some of these proteins have a known role in cancer progression and invasion, an interactions analysis did not reveal known biological processes or significant interactions between them that may underlie correlation to response to radiation.

## 4 DISCUSSION

Currently, there is no reliable method of predicting response to neoadjuvant chemoradiation in gastroesophageal cancer. Standard treatment protocols for esophageal gastroesophageal cancer include either neoadjuvant chemoradiation or perioperative chemotherapy, and it is not entirely clear whether one of these approaches is preferable for gastroesophageal junction tumors. The current ongoing prospective randomized ESOPEC trial^16^ may indicate which approach is preferred. In the current study we aimed to reveal whether there is a biomarker profile that may predict improved response to chemoradiation. Such a signature could potentially be used for selecting a personalized neoadjuvant therapy protocol.

We identified 2885 proteins using proteomic analysis of 35 patients divided into two cohorts upon recurrence pattern. In the favorable prognosis group, we identified 87 differentially enriched proteins in patient who were treated with neoadjuvant chemoradiation. A STRING analysis of these 87 proteins revealed significant enrichment of proteins related to cellular respiration, mitochondrial ATP synthesis coupled electron transport, organophosphate metabolic processes and mitochondrial respiratory chain complexes assembly.

Mitochondrial oxidative phosphorylation is upregulated in most types of cancer cells and is currently being investigated as a potential target for cancer treatment.^17^, ^18^, ^19^ One group of differentially enriched proteins we found in this study were NDUF proteins which belong to mitochondrial respiratory Complex I (including NDUFS2, NDUFA1, NDUFA9, NDUFB5, NDUFA12). This group of proteins is the largest component of the mitochondrial oxidative phosphorylation system. Mitochondrial Complex I is known to play a significant role in cancer cells resistance and metastatic potential.^20^ Complex I proteins allow cancer cells to adapt to hypoxia while continuing to proliferate.^21^ Our findings regarding Complex 1 protein are supported by a previous study that demonstrated how esophageal adenocarcinoma patients with a poor pathological response to neoadjuvant chemoradiation, had an increase in oxidative phosphorylation activity.^22^ This study also demonstrated that resistant esophageal adenocarcinoma cells can effectively switch between glycolysis and oxidative phosphorylation and were thus less effected by ionizing radiation.^22^ Furthermore, in a different study, inhibition of Complex I proteins was also shown to reprogram the immune microenvironment of esophageal cancer cells, in the setting of a clinical trial.^23^ This was achieved utilizing a common drug for diabetes mellitus—Metformin, which inhibits the function of Complex 1 proteins.^24^ These findings also reveal the role of Complex I mitochondrial proteins in the survival of chemoradiation resistant esophageal cancer cells and suggest that further research efforts should focus on Complex 1 inhibition to improve response to neoadjuvant treatment for esophageal cancer.

Another group of differently enriched interacting proteins in the favorable prognosis group were RAB39A, RAB4A and RAB5A. These proteins belong to the Ras Associated Binding (RAB) GTPase family of proteins. RAB proteins has an important role in intracellular vesicle trafficking and controls a large number of signaling cascades. RAB proteins were shown to regulate the induction of a gene signature for chemotherapy and radiation resistance in breast cancer.^25^ RAB proteins are also regulating apoptotic pathways which have a significant role in cancer progression and may serve as potential therapeutic targets.^26^ Proteins of the RAB family were shown to be markers for radio‐resistance in esophageal cancer,^27^ as well as potential targets for radio‐sensitization of rectal cancers.^28^ Considering this evidence and our findings it seems that following further validation, RAB proteins may serve as biomarkers for response to neoadjuvant chemoradiation in esophageal cancer.

Our analysis identified 11 significantly enriched proteins in the poor prognosis group. Some of these proteins are known to play a significant role in biologically related cancers survival. One example is DSG1, a type of desmosomal cadherin, known to be associated with decreased survival in head and neck squamous cell carcinoma.^29^ Another example is RAb11‐FIP2 which was shown to be increased in an immunohistochemical analysis of 86 gastric cancer patients and was closely correlated with nodal metastasis.^30^ Though each of these proteins may be a potential biomarker for unfavorable response to chemoradiation, but our analysis did not identify any common biological process or interaction between these proteins.

Study limitations account for heterogenous chemotherapeutic protocols that may modify proteomic signature of the resected tumor. Nevertheless, a substantial part received fluoropyrimidine and platinum and the others taxane and platinum. We employed proteomic analysis of surgical specimens following neoadjuvant treatment, that may have altered baseline proteomic landscape. Most importantly, this is a preliminary report, that warrants further validation. Following such validation, the proteins we identified may serve as biomarkers for favorable response to radiation in gastroesophageal cancer. Assuming our findings are indeed validated, future studies that will prospectively employ baseline and posttreatment samples may provide additional insights for esophageal cancer patients stratification.

## 5 CONCLUSIONS

In conclusion, our study revealed a potential predictive proteomic signature to neoadjuvant chemoradiation in patients with esophageal and gastroesophageal adenocarcinoma. In a cohort of patients with a favorable response to neoadjuvant chemoradiation, we have identified enrichment of pathways related to Complex I mitochondrial proteins and intracellular vesicular trafficking. These pathways and proteins may be important biomarkers for response to radiation therapy in esophageal cancer.

Further studies are warranted to confirm whether these pathways and proteins may serve as a surrogate biomarker for patient selection. Thus, Neoadjuvant chemoradiation may be administered to appropriate patients in an era of optional perioperative chemotherapy as an alternative mode of neoadjuvant therapy for GEJ tumors.

## CONFLICT OF INTEREST

The authors have stated explicitly that there are no conflicts of interest in connection with this article.

## AUTHOR CONTRIBUTIONS

All authors had full access to the data in the study and take responsibility for the integrity of the data and the accuracy of the data analysis. *Conceptualization*, I.B.A.; *Data Curation*, O.Z., B.B., Y.K.; *Formal Analysis*, O.Z., T.G.L., R.H., *Funding Acquisition*, I.B.A.; *Investigation*, O.Z.; *Methodology*, T.G.L., R.H., I.B.A.; *Project Administration*, T.G.L., I.B.A.; *Resources*, T.G.L., I.B.A.; *Software*, T.G.L.; *Supervision*, T.G.L., R.H., I.B.A., H.K.; *Validation*, B.B., Y.K., I.B.A., H.K.; *Visualization*, O.Z.; *Writing‐Original Draft*, O.Z.; *Writing‐Review & Editing*, O.Z., R.H., I.B.A., H.K.

## ETHICAL STATEMENT

The protocol was approved by the IRB committee (RMC‐0790‐16), as this is a retrospective study, no patient consent was required by the IRB.

## Data Availability

Clinical data are stored in an institutional database and will be shared upon request to the corresponding author. The mass spectrometry proteomics data have been deposited to the ProteomeXchange Consortium via the PRIDE^31^ partner repository with the dataset identifier PXD025192. Project Name: Proteomic Analysis to Identify Markers for Response to Neoadjuvant Treatment in Esophageal and Gastroesophageal Cancer Project accession: PXD025192 Project DOI: Not applicable Reviewer account details: Username: reviewer_pxd025192@ebi.ac.uk Password: 0ntF4F5s
